# Advancing immunotherapy for melanoma: the critical role of single-cell analysis in identifying predictive biomarkers

**DOI:** 10.3389/fimmu.2024.1435187

**Published:** 2024-07-04

**Authors:** Ru He, Jiaan Lu, Jianglong Feng, Ziqing Lu, Kaixin Shen, Ke Xu, Huiyan Luo, Guanhu Yang, Hao Chi, Shangke Huang

**Affiliations:** ^1^ Clinical Medical College, Southwest Medical University, Luzhou, China; ^2^ Department of Pathology, The Affiliated Hospital of Guizhou Medical University, Guiyang, China; ^3^ Department of Art and Design, Shanghai Institute of Technology, Shanghai, China; ^4^ Department of Oncology, Chongqing General Hospital, Chongqing University, Chongqing, China; ^5^ Department of Specialty Medicine, Ohio University, Athens, OH, United States; ^6^ Department of Oncology, The Affiliated Hospital, Southwest Medical University, Luzhou, Sichuan, China

**Keywords:** single-cell analysis, melanoma, immunotherapy, predictive biomarkers, TME, ICI

## Abstract

Melanoma, a malignant skin cancer arising from melanocytes, exhibits rapid metastasis and a high mortality rate, especially in advanced stages. Current treatment modalities, including surgery, radiation, and immunotherapy, offer limited success, with immunotherapy using immune checkpoint inhibitors (ICIs) being the most promising. However, the high mortality rate underscores the urgent need for robust, non-invasive biomarkers to predict patient response to adjuvant therapies. The immune microenvironment of melanoma comprises various immune cells, which influence tumor growth and immune response. Melanoma cells employ multiple mechanisms for immune escape, including defects in immune recognition and epithelial-mesenchymal transition (EMT), which collectively impact treatment efficacy. Single-cell analysis technologies, such as single-cell RNA sequencing (scRNA-seq), have revolutionized the understanding of tumor heterogeneity and immune microenvironment dynamics. These technologies facilitate the identification of rare cell populations, co-expression patterns, and regulatory networks, offering deep insights into tumor progression, immune response, and therapy resistance. In the realm of biomarker discovery for melanoma, single-cell analysis has demonstrated significant potential. It aids in uncovering cellular composition, gene profiles, and novel markers, thus advancing diagnosis, treatment, and prognosis. Additionally, tumor-associated antibodies and specific genetic and cellular markers identified through single-cell analysis hold promise as predictive biomarkers. Despite these advancements, challenges such as RNA-protein expression discrepancies and tumor heterogeneity persist, necessitating further research. Nonetheless, single-cell analysis remains a powerful tool in elucidating the mechanisms underlying therapy response and resistance, ultimately contributing to the development of personalized melanoma therapies and improved patient outcomes.

## 1 Introduction

Melanoma, a type of malignant skin cancer originating from melanocytes, poses a serious health risk. Once it metastasizes, it quickly disseminates throughout the body, resulting in a significantly poor prognosis (1). At present, its treatment methods include surgery, radiation therapy, immunotherapy et al. Immunotherapy, a pioneering approach, leverages the body’s immune system to combat cancer, offering hope when conventional treatments fall short. Among the most assertive and promising techniques for advanced melanoma treatment is ICI, which aids in boosting patient survival rates. However, advanced melanoma continues to exhibit a high mortality rate (2). Consequently, identifying non-invasive and reliable biomarkers to differentiate patients who will benefit from adjuvant therapy from those who will not is urgently needed. In targeted therapy and immunotherapy, there is a significant improvement in objective treatment efficacy and overall survival compared to traditional methods, but the emergence of drug resistance has had an impact on these positive outcomes,and tumor heterogeneity is playing a significant role in drug resistance (3, 4). In this context, single-cell technology has become an important information acquisition platform that can decipher complex clone relationships and potentially reveal the factors contributing to intratumoral heterogeneity with respect to MAPKi and ICI therapy resistance (5). Moreover, it plays an important role in analyzing the TIME, cancer progression, and immunotherapy response (6).

## 2 The immune microenvironment of melanoma

In the melanoma microenvironment, a diverse array of immune cells can be found. These include CD8+ T cells, CD4+ T cells, regulatory T cells (Tregs), natural killer (NK) cells, macrophages, and dendritic cells (DCs), along with several other types. These cells influence tumor growth and immune responses by secreting cytokines and chemokines, as well as interacting directly with tumor cells (7–9). As melanoma progresses, the acquisition of immune suppression in the environment and changes in endogenous pathways allow for immune escape. The main mechanisms of immune escape in melanoma include immune recognition defects, immune checkpoint receptors, and epithelial-mesenchymal transition (EMT) (10). Melanoma cells counteract antigen recognition and immune system stimulation through various strategies. Kimberly R. Jordan and her team have observed that the melanoma environment sees an accumulation of myeloid-derived suppressor cells (MDSCs) and Tregs, resulting in immune suppression. In patients with advanced melanoma, the populations of CD14+ and CD14− MDSCs in peripheral blood are significantly increased compared to healthy donors, and the frequency of MDSCs is significantly correlated with the frequency of Tregs, indicating a significant increase and interrelation of immunosuppressive cells in patients with advanced melanoma (correlation between MDSCs and Tregs) (11). Regulatory T cells (Tregs) can produce immunosuppressive cytokines, such as IL-10, TGF-β, and IL-35. These cytokines can inhibit the functions of effector T cells, dendritic cells, and other immune cells, thereby reducing their ability to generate immune responses (12, 13). They can also upregulate immunosuppressive molecules, such as PD-1(programmed cell death protein 1) and CTLA-4 (cytotoxic T-lymphocyte-associated protein 4), further inhibiting anti-tumor immune responses by suppressing the antigen-presenting capability of antigen-presenting cells (such as macrophages). Additionally, Tregs contribute to resistance to immune checkpoint inhibitors. Their presence in the tumor microenvironment can lead to acquired resistance by upregulating compensatory immune escape mechanisms (14). Moreover, IL-10 and IDO further reduce the activity of NK cells, CD4+, and CD8+ lymphocytes against melanoma (11). The dysregulation of immune checkpoint receptors, such as PD-1 and CTLA-4, is a critical mechanism by which melanoma escapes the immune system (15). Tumor cells can inhibit the function of cytotoxic T cells by abnormally expressing high levels of PD-L1, the ligand for PD-1. This PD-1/PD-L1 pathway forms the foundation for many immunotherapies. Immune checkpoint inhibitors, which are primarily used to treat melanoma, target two main proteins: PD-1 with monoclonal antibodies like nivolumab and pembrolizumab, and CTLA-4 with ipilimumab (15). These therapies assist in reinstating the immune system’s capability to target and destroy cancer cells. The process of epithelial-mesenchymal transition (EMT) is essential for metastatic cells to successfully colonize distant organs and is a significant mechanism of melanoma malignancy. EMT transcription factors (EMT-TFs) like SNAI1/2, ZEB1/2, and TWIST are responsible for regulating phenotype transitions, which are vital for melanoma progression. Additionally, these factors can influence antigen presentation, MHC I expression, and immune checkpoint regulation, thereby facilitating immune evasion (16, 17). These mechanisms allow melanoma cells to survive and proliferate under immune surveillance, impacting treatment outcomes and patient prognosis. Studying these immune escape mechanisms is crucial for developing new treatment strategies and improving the clinical management of melanoma patients.

## 3 The principles and revolutionary impact of single-cell analysis technology

Single-cell analysis encompasses both technology and methodology aimed at investigating the characteristics, functions, and behaviors of individual cells to attain a deeper comprehension of their diversity and complexity across various dimensions such as morphology, biochemistry, and genetics (18). This field comprises diverse techniques such as single-cell RNA sequencing, proteomics, and DNA sequencing. Recently, single-cell sequencing (sc-seq) technologies have primarily concentrated on comparing specific compartments within individual cells, such as the genome, transcriptome, epigenome, and proteome, to elucidate differences between cell populations and assess their heterogeneity (19). This comparative approach allows for the identification of rare cell populations, such as highly reactive immune cells within the tumor microenvironment (TME), and facilitates lineage tracking and developmental relationship analysis, including the diversity of lymphocyte fates. Moreover, particularly in the context of sc-RNAseq, exploring co-expression patterns of genes at the single-cell level enables the detection of co-regulated gene modules and regulatory networks associated with cell function and specification heterogeneity (20, 21). Single-cell sequencing also holds significant promise in immuno-oncology, offering insights into immune infiltration, trajectory inference, functional enrichments, and TCR repertoire analysis, thereby fostering opportunities for personalized medicine (22, 23).

## 4 Utilization & challenges of single-cell analysis technology in biomarker discovery for melanoma

The search for biomarkers is intricately influenced by the complexity and dynamism of the tumor microenvironment and the interplay between the immune system and cancer cells. Conventional analysis methods, reliant on large sample sizes, fall short in capturing this complexity, prompting a shift towards single-cell analysis. In melanoma and other cancer types, single-cell transcriptomics has emerged as a pivotal tool, facilitating the discovery of multidimensional biomarker signatures linked to both immune therapy response and resistance. This advancement holds promise in shaping the next generation of immune therapies aimed at enhancing cancer patient survival rates. Leveraging these techniques not only enhances our comprehension of immune therapies like immune checkpoint inhibitors but also sheds light on the tumor microenvironment’s role in immune therapy. Presently, biomarker identification primarily hinges on batch expression data analysis, necessitating endeavors to scrutinize cell type-specific gene expression features as plausible biomarkers. The application of single-cell technology in immuno-oncology research has demonstrated potential in delineating tumor microenvironment characteristics that influence immune therapy response and resistance across various cancer types, including melanoma. For instance, the amalgamation of scRNA-seq and TCR-seq has revealed the presence of functionally impaired CD8 T cells forming a proliferation hub within human melanoma, alongside a notable accumulation of functionally impaired T cells linked to tumor recognition (24). Moreover, experiments employing extensive RNA sequencing have identified B cell markers as the most differentially expressed genes between responders and non-responders in tumors. These findings offer valuable insights into the potential roles of B cells and tertiary lymphoid structures in immune checkpoint blockade (ICB) therapy response, thus holding significant implications for biomarker development and therapeutic targeting (25). Nevertheless, it is imperative to acknowledge that single-cell and single-nucleus RNA sequencing fall short in bridging the gap between RNA and protein expression, likely due to technical capture challenges (such as transcription capture/dropout) and biological intricacies (including translation barriers, post-translational effects, RNA degradation kinetics, or protein transport to the cell surface) (26, 27), whereas multi-omic CITE-seq holds promise in bridging this gap (28).

Besides above, the accuracy and reliability of melanoma biomarker discovery still need improvement, and single-cell analysis requires advancements and optimizations in technology and methods.

Briefly, the future directions include the following:

1) Mass Cytometry (CyTOF):

Capable of measuring multiple protein markers at the single-cell level, improving the sensitivity [Integration of online desalting techniques with dual-spray mass spectrometry has improved the detection sensitivity of cell surface proteins (29)] and throughput of marker detection [A novel Zr-NMOF-based mass tag has been developed, which provides fivefold signal amplification and allows for the detection of low-abundance antigens, improving sensitivity and multiplexing capability (30)].

Development of new antibodies and tags [Development of new metal-containing tags using click chemistry has enabled the generation of highly sensitive and specific reagents for proteomics and glycomics applications (31)] to increase the number and range of detectable proteins, along with improvements in data processing and analysis methods [Comparison of clustering methods for high-dimensional CyTOF data has shown that algorithms like FlowSOM and PhenoGraph are effective for defining cell populations, enhancing the efficiency of data analysis (32)].

2) Spatial Transcriptomics:

Combines single-cell sequencing with spatial information from tissue samples to provide gene expression maps of cells in their native tissue environment.

Improvements include enhancing spatial resolution [BayesSpace (33), DIST (34)] and data integration capabilities [SPOTlight (35), PRECAST (36) and SpatialScope (37)].

Development of higher resolution imaging and sequencing technologies, as well as advanced data analysis tools to integrate spatial and single-cell data [As Benchmarking Computational Integration Methods: Evaluates various methods for integrating spatially variable and highly variable genes from spatial transcriptomics data to improve clustering performance (38)].

3) Single-Cell Epigenomics (e.g., single-cell ATAC-seq):

Used to analyze chromatin accessibility [In cancer (39), Leukemia (40) and Schizophrenia (41)] and epigenetic modifications at the single-cell level.

Technological advancements aim to improve data quality and analysis precision.

Improvements to single-cell ATAC-seq techniques to better understand how chromatin state regulates gene expression.

4) Multi-Omics Data Integration:

By integrating single-cell RNA sequencing, DNA sequencing, proteomics, and epigenomics data, a more comprehensive understanding of cell states and functions can be achieved.

The key is to develop effective data integration and analysis methods (42).

Development of new computational and bioinformatics tools to efficiently integrate and interpret multi-omics data, revealing complex biological mechanisms. Various computational and bioinformatics tools, such as LIBRA and Cobolt, have been developed to address the challenges of multi-omics data integration. These tools improve the accuracy of data analysis and enhance the biological insights gained from multi-omics studies (43).

Additionally, the continuous development of artificial intelligence (AI) technologies brings many possibilities to the field of single-cell sequencing, injecting fresh vigor. Utilizing advanced machine learning and AI techniques to analyze single-cell data can improve the efficiency and accuracy of data processing and pattern recognition. Development of AI algorithms specifically for single-cell analysis can enhance data interpretation capabilities, particularly in identifying rare cell types and states.

Through advancements in these technologies and methods, the application of single-cell analysis in melanoma biomarker discovery will become more accurate and reliable, thereby promoting the development of personalized treatment and precision medicine.

## 5 Discovery of predictive biomarkers

In the earlier discussion, single-cell analysis techniques can be employed to examine cellular composition and status, analyze gene profiles, identify cell markers, and uncover novel melanoma markers. This, in turn, offers novel avenues for the diagnosis, treatment, and prognosis of melanoma. Established biomarkers encompass tumor-related antibodies, circulating biomarkers, cell markers, and specific genetic features.

### 5.1 Circulating biomarkers

Circulating biomarkers encompass a variety of detectable and quantifiable molecules, including DNA, RNA, proteins, and immune cells, which are released into the bloodstream and can serve as indicators of disease status (44). These biomarkers exhibit significant potential in predicting treatment response, facilitating diagnosis, and assessing prognosis. Blood-based liquid biopsy has garnered increasing interest due to its non-invasive and reliable nature. Despite the identification of numerous circulating biomarkers in both preclinical research and clinical settings, only few have received approval from FDA for clinical application. Consequently, comprehensive investigation into blood-based biomarkers holds considerable importance for melanoma patients undergoing immune checkpoint inhibitor (ICI) therapy, potentially paving the way for innovation in personalized medicine.

Researchers conducted single-cell RNA expression and protein sequencing (REAP-Seq) on longitudinally collected tumor and peripheral blood mononuclear cell (PBMC) samples before and after one cycle of immune checkpoint blockade (ICB). Their findings revealed that the abundance of tumor-infiltrating B cell clones had a prognostic impact on overall survival, with patients harboring a higher number of B cell clones in the tumor exhibiting improved survival prognosis post-ICB treatment. Furthermore, the CD14+ monocyte subset was implicated in a favorable response to ICB among melanoma patients (45). Single-cell analysis unveiled a specific monocyte phenotype (CD14[+]CD16 (–)CD33[+]HLA-DR[hi]) capable of predicting the response to PD-1 immunotherapy. While certain bone marrow cell populations (e.g., CD33[low]CD11b[+]HLA-DR[lo]) displayed no disparity in frequency between responders and non-responders during treatment, responsive patients showcased classical monocytes (CD14[+]CD16[−]) expressing more migration and activation markers (e.g., ICAM-1 and HLA-DR), suggesting their involvement in the immune response during PD-1 immunotherapy. Moreover, a decrease in pre-treatment T cell frequency correlated with treatment response and survival rate. Single-cell analysis demonstrated that CD8+ T cells exhibited enhanced cytolytic function and reduced immature phenotype during treatment. Despite gene expression not always mirroring protein levels, blood analysis of multiple cancer patients evidenced a correlation between immunotherapy and reduced frequency of immature-like CD8+ T cells, indicative of potentially improved treatment outcomes. Thus, specific CD8+ T cells and their markers in the blood hold promise as potential indicators for predicting immune therapy response (46).

### 5.2 Autologous (tumor-associated) antibodies

Tumor-specific antibodies have been the subject of extensive research in recent years. NY-ESO-1, a cancer/testis antigen, is expressed in a subset of melanoma patients. A 2016 study demonstrated that ipilimumab enhances the immune response against this antigen, and such antibody response correlates with the predictive value of ipilimumab treatment (47). Building on this, Fässler, Mirjam, and their colleagues conducted a cohort experiment hypothesizing that pre-existing antibodies against more widespread antigens might correlate with the clinical outcomes of melanoma patients undergoing PD-1/PD-L1 and CTLA4 treatment. Their experimental findings indicated that responders exhibited significantly stronger absorbance signals against NY-ESO-1, MelanA/MART1, TRP1/TYRP1, and TRP2/TYRP2, and these stronger signals were significantly associated with treatment response. Hence, these antibodies hold potential as novel biomarkers that could benefit metastatic melanoma patients (48). However, a recent study refuted this conclusion, finding no significant difference in serum levels of tumor-related antibodies between responders and non-responders, even across different overall survival groups (49). Further investigation is warranted to ascertain the potential of tumor-related antibodies as biomarkers. Moreover, some literature has highlighted dual-specificity antibodies that enhance the targeting effect and cytotoxicity of malignant melanoma by simultaneously targeting two antigens in tumor and immune cells, thus reducing drug resistance and offering a clinical treatment option (50, 51). Despite extensive recent research on specific antibodies, there is limited literature on the utilization of scRNA-seq techniques to aid in the study of predictive antibodies. scRNA-seq can unveil the cellular origin, expression patterns, and interactions of these autoantibodies with other components in the tumor microenvironment. For instance, researchers identified tumor-related NK cells via single-cell analysis, revealing impaired anti-tumor function, which correlates with poor prognosis and resistance to immunotherapy, potentially linked to autoantibody production. Additionally, single-cell analysis can elucidate changes in systemic immunity during tumor progression; researchers identified an adaptive NK cell subgroup characterized by upregulation of specific pro-inflammatory cytokines and MHC class II genes in the peripheral blood of cancer patients (52). This nuanced understanding at the cellular level can facilitate the discovery and validation of autoantibodies as predictive biomarkers. Single-cell analysis offers insights into the expression pattern and variation of tumor cell surface antigens, aiding in the design and screening of more specific and effective tumor antibodies. Therefore, further exploration of single-cell analysis in studying autoantibodies is warranted.

### 5.3 Genetic characteristics

In recent years, many studies on melanoma cells using single-cell technology have indicated that certain gene expressions are associated with the metastasis and prognosis of tumors (**Table 1**).

**Table 1:** This table provides some key findings of gene expressions are associated with the metastasis and prognosis of melanoma.

Study	Key Findings	Reference
Durante et al.	- Identified EIF1AX and SF3B1 gene mutations as potential predictive biomarkers associated with Class 1 UM - Genomic alterations and increased aneuploidy in Class 2 tumors associated with an immunosuppressive microenvironment - LAG3 emerged as a major exhaustion marker in UM, acting synergistically with the immune checkpoint receptor PD1	(53)
Zhang et al.	- Identified gene amplification on chromosome 4 and differentially expressed genes potentially associated with immunotherapy resistance - Enriched pathways related to EGFR and the cell cycle	(54)
Liu et al.	- Established glycosylation-related gene (GRG) features through the integration of extensive RNA-seq and scRNA-seq data - Identified AUP1 as a key gene affecting UM cell survival, proliferation, and invasion - Gene feature risk scoring system (GCNS) identified as an important prognostic factor for UM	(55)
Bakr et al.	- Demonstrated that melanoma cells expressing CHRNA1 are predominantly metastatic and exhibit high expression levels of CHRNB1, CHRNG, and genes associated with myogenesis/cell cycle	(56)
Huang et al.	- Treg cells become paradoxical contributors to immune evasion. 29 genes significantly associated with melanoma prognosis were identified among the top 200 marker genes in the C2 TIGIT+ Treg cell subset. Subsequently, employing the LASSO method, the gene pool was refined, strategically narrowing down the candidate genes to a set of six key marker genes (MALAT1, TTC39C, TNFRSF4, GBP5, B2M, and GBP2) as protective genes. - In C3 TNFRSF18+ Treg cells, TNFRSF4, CTLA4, IL2RA, GBP2, PSMB9, GADD45A, SH3BGRL, NAMPT, and PSMA2 were identified as protective factors (HR <1), while NDUFA13, CALM3, and PGAM1 were identified as high-risk factors (HR >1).	(57)
Wang et al.	- Utilized network embedding analysis of single-cell sequencing data to identify hub genes ETS1, TP53, E2F1, and GATA3 associated with melanoma	(58)
Bakr et al.	- Identified a melanoma prognostic signature composed of 45 genes (MPS_45) - MPS_45 demonstrated significant association with survival in TCGA-SKCM and three other melanoma datasets, independently predicting melanoma patient prognosis with high potential	(59)
Xie et al.	- Established an m7G gene feature for predicting survival and clinical outcomes in uveal melanoma (UVM) patients using single-cell analysis, weighted gene co-expression network analysis (WGCNA), and Lasso-Cox regression <br> - Identified the gene phosphoprotein membrane anchor 1 (PAG1) as most closely associated with patient prognosis	(60)

### 5.4 Cellular biomarkers

**Figure 1:**
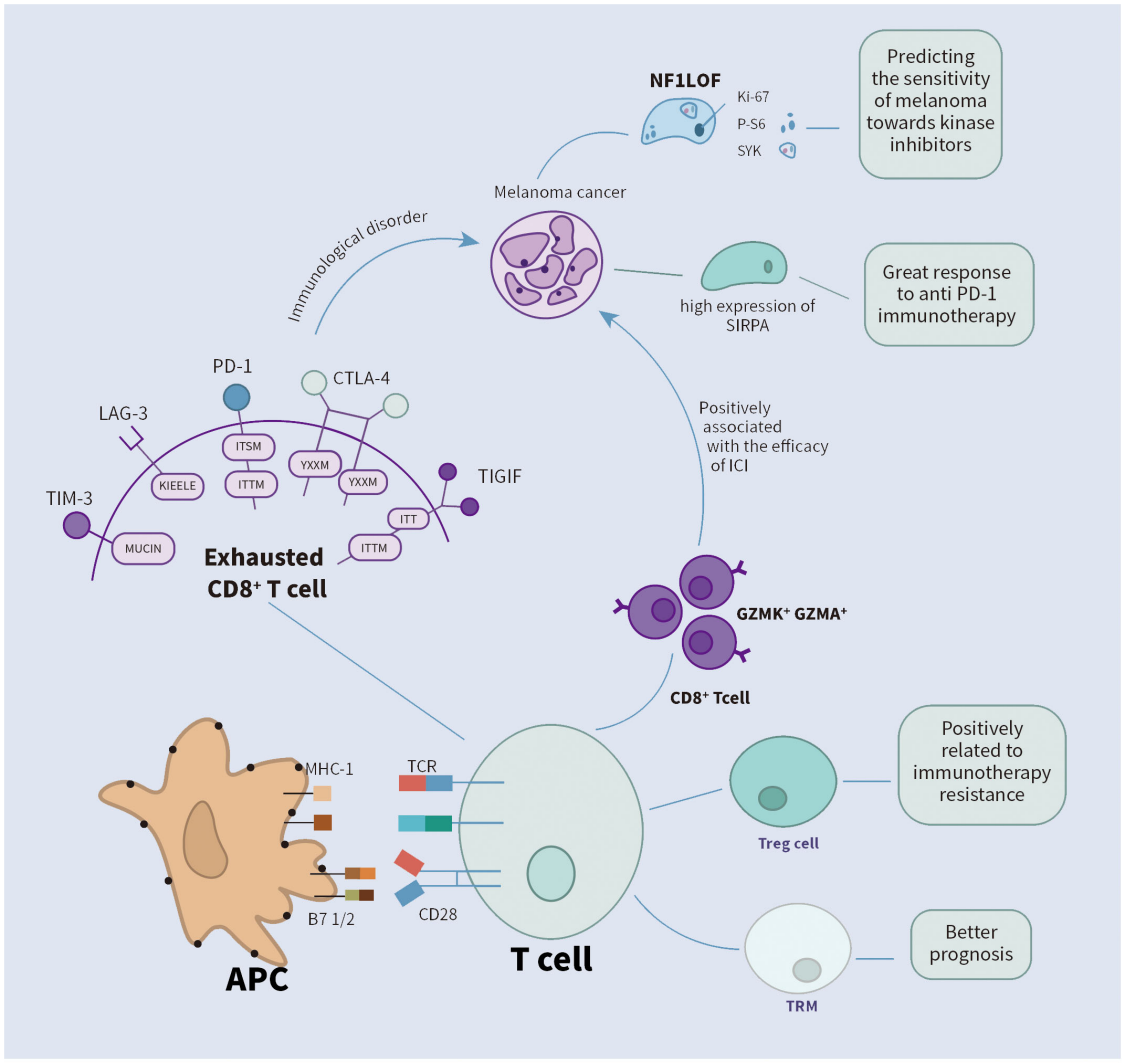
The role of cell markers identified by single-cell analysis in the immunotherapy of melanoma.

scRNA-seq has been utilized to examine the composition of tumor-immune cells in melanoma. Within the tumor, CD4 and CD8 T cells display distinct transcriptional profiles, revealing a gradual shift from initial effector states to dysfunctional T cell states (24, 61). CD8 T cells that are functionally impaired, exhibiting elevated levels of immunosuppressive molecules like LAG-3 and PD-1, are strongly linked to tumor malignancy (62). In melanoma research, scRNA-seq technology has aided in identifying various T cell subsets, such as CD8+ T cells, CD4+ T cells, Tregs, and NK cells are essential in the antitumor immune response. Their distinct phenotypes and functional states can act as biomarkers to predict how patients will respond to immunotherapy. For instance, research indicates that CD8+ T cells display varying degrees of dysfunction in the melanoma microenvironment, a phenomenon known as T cell exhaustion (63). Inhibitory receptors, including PD-1, CTLA-4, TIM-3, TIGIT, and LAG3, are commonly expressed by exhausted T cells (64, 65) (**Figure 1**). Although they express cytotoxic-related genes like IFNG, GZMB, and PRF1, indicating an active effector state, exhausted T cells show diminished or minimal expression of IL-2, tumor necrosis factor-alpha (TNF-α), and T-box transcription factor (TBX21). Notably, a minor subset of these exhausted CD8+ T cells exhibits elevated levels of MKi67, a marker indicating active proliferation, implying that T cell proliferation might precede the exhaustion process (66). scRNA-seq technology has identified a distinct subset of CD8+ T cells that express high levels of GZMK instead of GZMB. These GZMK+ T cells either lack or have low levels of exhaustion-related markers and have been detected in various tumor types, including melanoma. The prevalence of this GZMK+ T cell subset suggests it may play a significant role in tumor immunity. Indeed, a higher ratio of GZMK+ CD8+ T cells to exhausted T cells is positively correlated with improved survival rates in patients with non-small cell lung cancer and is linked to better responses to immune checkpoint inhibitors in melanoma patients (**Figure 1**). Through the utilization of scRNA-seq technology, researchers have also been able to track the clonal dynamics of T cells, including clonal expansion, tendencies of transcriptional state, enrichment in specific tissues, and propensity to migrate to other tissues. These insights aid in understanding the dynamic changes of T cells in the tumor microenvironment and may unveil new therapeutic targets (6). In the research on acral melanoma, it was found that the quantity of Treg cells is more significant in AM (advanced melanoma) compared to CM (conventional melanoma). And a greater abundance of Tregs is linked to increased resistance to immune therapy (54). Tissue-resident memory T cells (TRM) are distinct T cell populations that remain in peripheral tissues, particularly the skin. By examining scRNA-seq datasets from human melanoma, researchers have developed a TRM signature that effectively indicates the presence of tissue-resident memory T cells in melanoma patients. TRM infiltration in melanoma correlates with extended overall survival and increased quantities of T cells, NK cells, M1 macrophages, and memory B cells. This finding suggests that the presence of TRM could signify a more active TME, potentially enhancing patient outcomes (**Figure 1**) (67). Single-cell analysis reveals that NF1LoF (neurofibromatosis 1 loss of function) melanoma cells’ response to drugs is significantly associated with the inhibition of both Ki-67 (a marker of cell proliferation) and p-S6 (phosphorylation of ribosomal S6 protein, an effector molecule of the mTOR signaling pathway). Ki-67 is a nuclear protein associated with the cell cycle and commonly used as a marker for cell proliferation; p-S6 is a key factor in cell growth and survival. The inhibitory effects of these markers can serve as indicators for predicting the sensitivity of melanoma to kinase inhibitors. A study analyzed the expression of p-S6 and Ki-67 in three NF1LoF cell lines after treatment with MTX-216, pictilisib, trametinib, or ulixertinib, finding that all inhibitors reduced the expression of these two markers, and the inhibitory effects correlated with drug efficacy. Additionally, the study identified the importance of SYK kinase in NF1LoF melanoma cells, potentially becoming a novel predictive biomarker. The inhibitory effects of SYK and its relationship with mitochondrial function may help assess patients’ response to treatment. If SYK inhibitors can effectively suppress the growth and survival of melanoma cells, SYK activity levels may serve as a biomarker for predicting treatment outcomes (**Figure 1**) (68). Zhicheng Zhou et al. conducted integrated transcriptomic and proteomic analysis of emerging immunooncology targets in melanoma across multiple clinical cohorts receiving anti-PD-1 therapy, finding that patients with tumors carrying high expression of signal regulatory protein alpha (SIRPA) exhibited favorable responses to anti-PD-1 immunotherapy. SIRPA expression specific to tumors could serve as a more accurate biomarker compared to overall SIRPA expression. This is because overall expression might be influenced by signals from various cellular components present within the tumor, potentially causing confusion (**Figure 1**) (69).

## 6 Discussion

The TME in melanoma is characterized by a complex interplay between immune cells, cytokines, and tumor cells. Immune evasion mechanisms, including immune checkpoint receptor dysregulation and immune suppression by MDSCs and Tregs, contribute to tumor progression and therapy resistance (70, 71). Single-cell analysis elucidates these dynamics by identifying specific immune cell subsets and their functional states, offering potential biomarkers for therapy response prediction. Single-cell analysis can also construct a prognostic model for SKCM (skin cutaneous melanoma) based on relevant genetic markers in the immune microenvironment, such as necroptosis-related genes [NRGs (72) & CRG (73)], to help SKCM patients obtain precise clinical treatment strategies. Which opens up new avenues for patient prognosis evaluation.

Moreover, scRNA-seq facilitates the discovery of novel biomarkers beyond traditional approaches. For instance, circulating tumor cells (CTCs) and immune cells in the bloodstream harbor valuable information reflecting tumor dynamics and therapy response (74, 75). Recent studies have identified specific cell subsets, such as B cell clones and monocyte phenotypes, associated with improved survival and therapy response in melanoma patients undergoing ICI treatment (76). These findings underscore the prognostic significance of circulating biomarkers and emphasize the potential of scRNA-seq in personalized medicine.

Furthermore, scRNA-seq uncovers insights into cellular mechanisms underlying therapy response and resistance. For instance, the identification of functionally impaired CD8+ T cell subsets within the TME elucidates pathways of immune evasion and highlights potential targets for therapy intervention (62). Additionally, the characterization of TRM correlates with a more active tumor immune microenvironment and improved patient outcomes, suggesting TRM abundance as a prognostic marker (67).

Despite these advancements, challenges remain in translating single-cell analysis findings into clinical practice. Technical limitations, such as capturing RNA-protein expression discrepancies, and biological complexities, such as tumor heterogeneity, pose obstacles to biomarker validation and therapeutic targeting. To integrate the findings from single-cell analysis into clinical practice for personalized melanoma treatment, the following potential obstacles need to be overcome: the complexity of technology translation, the consistency of data interpretation, and the reliability of clinical validation. These obstacles need to be addressed through multidisciplinary collaboration and continuous technological development. Further research is needed to overcome these challenges and harness the full potential of single-cell analysis in melanoma immunotherapy.

In conclusion, single-cell analysis represents a revolutionary approach in advancing melanoma immunotherapy through the identification of predictive biomarkers, elucidation of therapy mechanisms, and characterization of the tumor immune microenvironment. By unraveling the intricacies of melanoma biology at the single-cell level, this technology holds promise for improving patient outcomes and shaping the future of precision oncology.

## Author contributions

RH: Data curation, Writing – original draft, Writing – review & editing. JL: Data curation, Project administration, Writing – original draft, Writing – review & editing. JF: Writing – original draft, Writing – review & editing. ZL: Writing – original draft, Writing – review & editing. KS: Writing – original draft, Writing – review & editing. KX: Writing – original draft, Writing – review & editing. HL: Writing – original draft, Writing – review & editing. GY: Supervision, Writing – original draft, Writing – review & editing. HC: Supervision, Writing – original draft, Writing – review & editing. SH: Supervision, Writing – original draft, Writing – review & editing.

## References

[1] Manola J Atkins M Ibrahim J Kirkwood J. Prognostic factors in metastatic melanoma: a pooled analysis of Eastern Cooperative Oncology Group trials. J Clin Oncol. (2000) 18:3782–93. doi: 10.1200/JCO.2000.18.22.3782 11078491

[2] Thoms KM. Advanced melanoma-A curable disease? Implications of systemic treatment on patients’ daily life. J Eur Acad Dermatol Venereol. (2023) 37:853–4. doi: 10.1111/jdv.19018 37052422

[3] Fu F Nowak MA Bonhoeffer S. Spatial heterogeneity in drug concentrations can facilitate the emergence of resistance to cancer therapy. PloS Comput Biol. (2015) 11:e1004142. doi: 10.1371/journal.pcbi.1004142 25789469 PMC4366398

[4] Saunders NA Simpson F Thompson EW Hill MM Endo-Munoz L Leggatt G. Role of intratumoural heterogeneity in cancer drug resistance: molecular and clinical perspectives. EMBO Mol Med. (2012) 4:675–84. doi: 10.1002/emmm.201101131 PMC3494067 22733553

[5] Fattore L Ruggiero CF Liguoro D Mancini R Ciliberto G. Single cell analysis to dissect molecular heterogeneity and disease evolution in metastatic melanoma. Cell Death Dis. (2019) 10:827. doi: 10.1038/s41419-019-2048-5 31672982 PMC6823362

[6] Ren X Zhang L Zhang Y Li Z Siemers N Zhang Z. Insights gained from single-cell analysis of immune cells in the tumor microenvironment. Annu Rev Immunol. (2021) 39:583–609. doi: 10.1146/annurev-immunol-110519-071134 33637019

[7] Wang J Peng C Dai W Chen X Meng J Jiang T. Exploring tumor immune microenvironment and its associations with molecular characteristics in melanoma. Front Oncol. (2022) 12:821578. doi: 10.3389/fonc.2022.821578 35530341 PMC9069107

[8] Tucci M Passarelli A Mannavola F Felici C Stucci LS Cives M. Immune system evasion as hallmark of melanoma progression: the role of dendritic cells. Front Oncol. (2019) 9:1148. doi: 10.3389/fonc.2019.01148 31750245 PMC6848379

[9] Willsmore ZN Harris RJ Crescioli S Hussein K Kakkassery H Thapa D. B cells in patients with melanoma: implications for treatment with checkpoint inhibitor antibodies. Front Immunol. (2020) 11:622442. doi: 10.3389/fimmu.2020.622442 33569063 PMC7868381

[10] Shirley CA Chhabra G Amiri D Chang H Ahmad N. Immune escape and metastasis mechanisms in melanoma: breaking down the dichotomy. Front Immunol. (2024) 15:1336023. doi: 10.3389/fimmu.2024.1336023 38426087 PMC10902921

[11] Jordan KR Amaria RN Ramirez O Callihan EB Gao D Borakove M. Myeloid-derived suppressor cells are associated with disease progression and decreased overall survival in advanced-stage melanoma patients. Cancer Immunol Immunother. (2013) 62:1711–22. doi: 10.1007/s00262-013-1475-x PMC4176615 24072401

[12] Stockis J Liénart S Colau D Collignon A Nishimura SL Sheppard D. Blocking immunosuppression by human Tregs in *vivo* with antibodies targeting integrin αVβ8. Proc Natl Acad Sci USA. (2017) 114:E10161–e10168. doi: 10.1073/pnas.1710680114 29109269 PMC5703296

[13] Viguier M Lemaître F Verola O Cho MS Gorochov G Dubertret L. Foxp3 expressing CD4+CD25(high) regulatory T cells are overrepresented in human metastatic melanoma lymph nodes and inhibit the function of infiltrating T cells. J Immunol. (2004) 173:1444–53. doi: 10.4049/jimmunol.173.2.1444 15240741

[14] Saleh R Elkord E. Treg-mediated acquired resistance to immune checkpoint inhibitors. Cancer Lett. (2019) 457:168–79. doi: 10.1016/j.canlet.2019.05.003 31078738

[15] Ott PA Hodi FS Robert C. CTLA-4 and PD-1/PD-L1 blockade: new immunotherapeutic modalities with durable clinical benefit in melanoma patients. Clin Cancer Res. (2013) 19:5300–9. doi: 10.1158/1078-0432.CCR-13-0143 24089443

[16] Caramel J Papadogeorgakis E Hill L Browne GJ Richard G Wierinckx A. A switch in the expression of embryonic EMT-inducers drives the development of Malignant melanoma. Cancer Cell. (2013) 24:466–80. doi: 10.1016/j.ccr.2013.08.018 24075834

[17] Terry S Buart S Tan TZ Gros G Noman MZ Lorens JB. Acquisition of tumor cell phenotypic diversity along the EMT spectrum under hypoxic pressure: Consequences on susceptibility to cell-mediated cytotoxicity. Oncoimmunology. (2017) 6:e1271858. doi: 10.1080/2162402X.2016.1271858 28344883 PMC5353930

[18] Forcato M Romano O Bicciato S. Computational methods for the integrative analysis of single-cell data. Brief Bioinform. (2021) 22:20–9. doi: 10.1093/bib/bbaa042 PMC7820847 32363378

[19] Sierant MC Choi J. Single-cell ssequencing in cancer: recent applications to immunogenomics and multi-omics tools. Genomics Inform. (2018) 16:e17. doi: 10.5808/GI.2018.16.4.e17 30602078 PMC6440661

[20] Algabri YA Li L Liu ZP. scGENA: A single-cell gene coexpression network analysis framework for clustering cell types and revealing biological mechanisms. Bioeng (Basel). (2022) 9:353. doi: 10.3390/bioengineering9080353 PMC9405199 36004879

[21] Vivian Li W Li Y. scLink: inferring sparse gene co-expression networks from single-cell expression data. Genomics Proteomics Bioinf. (2021) 19:475–92. doi: 10.1016/j.gpb.2020.11.006 PMC8896229 34252628

[22] Lei Y Tang R Xu J Wang W Zhang B Liu J. Applications of single-cell sequencing in cancer research: progress and perspectives. J Hematol Oncol. (2021) 14:91. doi: 10.1186/s13045-021-01105-2 34108022 PMC8190846

[23] Gohil SH Iorgulescu JB Braun DA Keskin DB Livak KJ. Applying high-dimensional single-cell technologies to the analysis of cancer immunotherapy. Nat Rev Clin Oncol. (2021) 18:244–56. doi: 10.1038/s41571-020-00449-x PMC8415132 33277626

[24] Li H van der Leun AM Yofe I Lubling Y Gelbard-Solodkin D van Akkooi ACJ. Dysfunctional CD8 T cells form a proliferative, dynamically regulated compartment within human melanoma. Cell. (2019) 176:775–789.e18. doi: 10.1016/j.cell.2018.11.043 30595452 PMC7253294

[25] Helmink BA Reddy SM Gao J Zhang S Basar R Thakur R. B cells and tertiary lymphoid structures promote immunotherapy response. Nature. (2020) 577:549–55. doi: 10.1038/s41586-019-1922-8 PMC8762581 31942075

[26] Vogel C Marcotte EM. Insights into the regulation of protein abundance from proteomic and transcriptomic analyses. Nat Rev Genet. (2012) 13:227–32. doi: 10.1038/nrg3185 PMC3654667 22411467

[27] Payne SH. The utility of protein and mRNA correlation. Trends Biochem Sci. (2015) 40:1–3. doi: 10.1016/j.tibs.2014.10.010 25467744 PMC4776753

[28] Lischetti U Tastanova A Singer F Grob L Carrara M Cheng PF. Dynamic thresholding and tissue dissociation optimization for CITE-seq identifies differential surface protein abundance in metastatic melanoma. Commun Biol. (2023) 6:830. doi: 10.1038/s42003-023-05182-6 37563418 PMC10415364

[29] Xu S Xue J Bai Y Liu H. High-throughput single-cell immunoassay in the cellular native environment using online desalting dual-spray mass spectrometry. Anal Chem. (2020) 92:15854–61. doi: 10.1021/acs.analchem.0c03167 33231067

[30] Dang J Li H Zhang L Li S Zhang T Huang S. New structure mass tag based on zr-NMOF for multiparameter and sensitive single-cell interrogating in mass cytometry. Adv Mater. (2021) 33:e2008297. doi: 10.1002/adma.202008297 34309916

[31] Allo B Lou X Bouzekri A Ornatsky O. Clickable and high-sensitivity metal-containing tags for mass cytometry. Bioconjug Chem. (2018) 29:2028–38. doi: 10.1021/acs.bioconjchem.8b00239 29733585

[32] Weber LM Robinson MD. Comparison of clustering methods for high-dimensional single-cell flow and mass cytometry data. Cytomet A. (2016) 89:1084–96. doi: 10.1002/cyto.a.23030 27992111

[33] Zhao E Stone MR Ren X Guenthoer J Smythe KS Pulliam T. Spatial transcriptomics at subspot resolution with BayesSpace. Nat Biotechnol. (2021) 39:1375–84. doi: 10.1038/s41587-021-00935-2 PMC8763026 34083791

[34] Zhao Y Wang K Hu G. DIST: spatial transcriptomics enhancement using deep learning. Brief Bioinform. (2023) 24:bbad013. doi: 10.1093/bib/bbad013 36653906

[35] Elosua-Bayes M Nieto P Mereu E Gut I Heyn H. SPOTlight: seeded NMF regression to deconvolute spatial transcriptomics spots with single-cell transcriptomes. Nucleic Acids Res. (2021) 49:e50. doi: 10.1093/nar/gkab043 33544846 PMC8136778

[36] Liu W Liao X Luo Z Yang Y Lau MC Jiao Y. Probabilistic embedding, clustering, and alignment for integrating spatial transcriptomics data with PRECAST. Nat Commun. (2023) 14:296. doi: 10.1038/s41467-023-35947-w 36653349 PMC9849443

[37] Wan X Xiao J Tam SST Cai M Sugimura R Wang Y. Integrating spatial and single-cell transcriptomics data using deep generative models with SpatialScope. Nat Commun. (2023) 14:7848. doi: 10.1038/s41467-023-43629-w 38030617 PMC10687049

[38] Yan L Sun X. Benchmarking and integration of methods for deconvoluting spatial transcriptomic data. Bioinformatics. (2023) 39:btac805. doi: 10.1093/bioinformatics/btac805 36515467 PMC9825747

[39] Liu Y. Clinical implications of chromatin accessibility in human cancers. Oncotarget. (2020) 11:1666–78. doi: 10.18632/oncotarget.v11i18 PMC7210018 32405341

[40] Moia R Terzi di Bergamo L Talotta D Bomben R Forestieri G Spina V. XPO1 mutations identify early-stage CLL characterized by shorter time to first treatment and enhanced BCR signalling. Br J Haematol. (2023) 203:416–25. doi: 10.1111/bjh.19052 37580908

[41] Bryois J Garrett ME Song L Safi A Giusti-Rodriguez P Johnson GD. Evaluation of chromatin accessibility in prefrontal cortex of individuals with schizophrenia. Nat Commun. (2018) 9:3121. doi: 10.1038/s41467-018-05379-y 30087329 PMC6081462

[42] Xiao C Chen Y Meng Q Wei L Zhang X. Benchmarking multi-omics integration algorithms across single-cell RNA and ATAC data. Brief Bioinform. (2024) 25:bbae095. doi: 10.1093/bib/bbae095 38493343 PMC10944570

[43] Gong B Zhou Y Purdom E. Cobolt: integrative analysis of multimodal single-cell sequencing data. Genome Biol. (2021) 22:351. doi: 10.1186/s13059-021-02556-z 34963480 PMC8715620

[44] Cappelletti V Appierto V Tiberio P Fina E Callari M Daidone MG. Circulating biomarkers for prediction of treatment response. J Natl Cancer Inst Monogr. (2015) 2015:60–3. doi: 10.1093/jncimonographs/lgv006 26063889

[45] Valpione S Campana LG Weightman J Salih Z Galvani E Mundra PA. Tumour infiltrating B cells discriminate checkpoint blockade-induced responses. Eur J Cancer. (2022) 177:164–74. doi: 10.1016/j.ejca.2022.09.022 36347135

[46] Khojandi N Connelly L Piening A Hoft SG Pherson M Donlin MJ. Single-cell analysis of peripheral CD8(+) T cell responses in patients receiving checkpoint blockade immunotherapy for cancer. Cancer Immunol Immunother. (2023) 72:397–408. doi: 10.1007/s00262-022-03263-9 35907015 PMC10306114

[47] Yuan J Adamow M Ginsberg BA Rasalan TS Ritter E Gallardo HF. Integrated NY-ESO-1 antibody and CD8+ T-cell responses correlate with clinical benefit in advanced melanoma patients treated with ipilimumab. Proc Natl Acad Sci USA. (2011) 108:16723–8. doi: 10.1073/pnas.1110814108 PMC3189057 21933959

[48] Fässler M Diem S Mangana J Hasan Ali O Berner F Bomze D. Antibodies as biomarker candidates for response and survival to checkpoint inhibitors in melanoma patients. J Immunother Cancer. (2019) 7:50. doi: 10.1186/s40425-019-0523-2 30786924 PMC6383238

[49] de Joode K Veenbergen S Kransse C Kortleve D Debets R Mathijssen RHJ. Suitability of tumor-associated antibodies as predictive biomarker for response to immune checkpoint inhibitors in patients with melanoma: a short report. J Immunother Cancer. (2023) 11:e006467. doi: 10.1136/jitc-2022-006467 36750254 PMC9906380

[50] Kim S Kim SA Nam GH Hong Y Kim GB Choi Y. *In situ* immunogenic clearance induced by a combination of photodynamic therapy and rho-kinase inhibition sensitizes immune checkpoint blockade response to elicit systemic antitumor immunity against intraocular melanoma and its metastasis. J Immunother Cancer. (2021) 9:e001481corr1. doi: 10.1136/jitc-2020-001481 33479026 PMC7825261

[51] Tang J Gong Y Ma X. Bispecific antibodies progression in Malignant melanoma. Front Pharmacol. (2022) 13:837889. doi: 10.3389/fphar.2022.837889 35401191 PMC8984188

[52] Tang F Li J Qi L Liu D Bo Y Qin S. A pan-cancer single-cell panorama of human natural killer cells. Cell. (2023) 186:4235–4251.e20. doi: 10.1016/j.cell.2023.07.034 37607536

[53] Durante MA Rodriguez DA Kurtenbach S Kuznetsov JN Sanchez MI Decatur CL. Single-cell analysis reveals new evolutionary complexity in uveal melanoma. Nat Commun. (2020) 11:496. doi: 10.1038/s41467-019-14256-1 31980621 PMC6981133

[54] Zhang C Shen H Yang T Li T Liu X Wang J. A single-cell analysis reveals tumor heterogeneity and immune environment of acral melanoma. Nat Commun. (2022) 13:7250. doi: 10.1038/s41467-022-34877-3 36433984 PMC9700682

[55] Liu J Zhang P Yang F Jiang K Sun S Xia Z. Integrating single-cell analysis and machine learning to create glycosylation-based gene signature for prognostic prediction of uveal melanoma. Front Endocrinol (Lausanne). (2023) 14:1163046. doi: 10.3389/fendo.2023.1163046 37033251 PMC10076776

[56] Bakr MN Takahashi H Kikuchi Y. CHRNA1 and its correlated-myogenesis/cell cycle genes are prognosis-related markers of metastatic melanoma. Biochem Biophys Rep. (2023) 33:101425. doi: 10.1016/j.bbrep.2023.101425 36654921 PMC9841360

[57] Huang W Kim BS Zhang Y Lin L Chai G Zhao Z. Regulatory T cells subgroups in the tumor microenvironment cannot be overlooked: Their involvement in prognosis and treatment strategy in melanoma. Environ Toxicol. (2024). doi: 10.1002/tox.24247 38530049

[58] Wang L Liu F Du L Qin G. Single-cell transcriptome analysis in melanoma using network embedding. Front Genet. (2021) 12:700036. doi: 10.3389/fgene.2021.700036 34290746 PMC8287331

[59] Bakr MN Takahashi H Kikuchi Y. Analysis of melanoma gene expression signatures at the single-cell level uncovers 45-gene signature related to prognosis. Biomedicines. (2022) 10:1478. doi: 10.3390/biomedicines10071478 35884783 PMC9313451

[60] Xie J Chen L Cao Y Ma C Zhao W Li J. Single cell sequencing analysis constructed the N7-methylguanosine (m7G)-related prognostic signature in uveal melanoma. Aging (Albany NY). (2023) 15:2082–96. doi: 10.18632/aging.v15i6 PMC10085590 36920166

[61] Topchyan P Xin G Chen Y Zheng S Burns R Shen J. Harnessing the IL-21-BATF pathway in the CD8(+) T cell anti-tumor response. Cancers (Basel). (2021) 13:1263. doi: 10.3390/cancers13061263 33809259 PMC7998696

[62] Hashimoto M Kamphorst AO Im SJ Kissick HT Pillai RN Ramalingam SS. CD8 T cell exhaustion in chronic infection and cancer: opportunities for interventions. Annu Rev Med. (2018) 69:301–18. doi: 10.1146/annurev-med-012017-043208 29414259

[63] Miller BC Sen DR Al Abosy R Bi K Virkud YV LaFleur MW. Subsets of exhausted CD8(+) T cells differentially mediate tumor control and respond to checkpoint blockade. Nat Immunol. (2019) 20:326–36. doi: 10.1038/s41590-019-0312-6 PMC6673650 30778252

[64] Lu J Veler A Simonetti B Raj T Chou PH Cross SJ. Five inhibitory receptors display distinct vesicular distributions in murine T cells. Cells. (2023) 12:2558. doi: 10.3390/cells12212558 37947636 PMC10649679

[65] Anderson AC Joller N Kuchroo VK. Lag-3, tim-3, and TIGIT: co-inhibitory receptors with specialized functions in immune regulation. Immunity. (2016) 44:989–1004. doi: 10.1016/j.immuni.2016.05.001 27192565 PMC4942846

[66] McLane LM Abdel-Hakeem MS Wherry EJ. CD8 T cell exhaustion during chronic viral infection and cancer. Annu Rev Immunol. (2019) 37:457–95. doi: 10.1146/annurev-immunol-041015-055318 30676822

[67] Jiang C Chao CC Li J Ge X Shen A Jucaud V. Tissue-resident memory T cell signatures from single-cell analysis associated with better melanoma prognosis. iScience. (2024) 27:109277. doi: 10.1016/j.isci.2024.109277 38455971 PMC10918229

[68] Abecunas C Whitehead CE Ziemke EK Baumann DG Frankowski-McGregor CL Sebolt-Leopold JS. Loss of NF1 in melanoma confers sensitivity to SYK kinase inhibition. Cancer Res. (2023) 83:316–31. doi: 10.1158/0008-5472.CAN-22-0883 PMC9845987 36409827

[69] Zhou Z Chen MM Luo Y Mojumdar K Peng X Chen H. Tumor-intrinsic SIRPA promotes sensitivity to checkpoint inhibition immunotherapy in melanoma. Cancer Cell. (2022) 40:1324–1340.e8. doi: 10.1016/j.ccell.2022.10.012 36332624 PMC9669221

[70] Haist M Stege H Grabbe S Bros M. The functional crosstalk between myeloid-derived suppressor cells and regulatory T cells within the immunosuppressive tumor microenvironment. Cancers (Basel). (2021) 13:210. doi: 10.3390/cancers13020210 33430105 PMC7827203

[71] Toor SM Elkord E. Therapeutic prospects of targeting myeloid-derived suppressor cells and immune checkpoints in cancer. Immunol Cell Biol. (2018) 96:888–97. doi: 10.1111/imcb.12054 29635843

[72] Song B Wu P Liang Z Wang J Zheng Y Wang Y. A novel necroptosis-related gene signature in skin cutaneous melanoma prognosis and tumor microenvironment. Front Genet. (2022) 13:917007. doi: 10.3389/fgene.2022.917007 35899194 PMC9309482

[73] Song B Chi H Peng G Song Y Cui Z Zhu Y. Characterization of coagulation-related gene signature to predict prognosis and tumor immune microenvironment in skin cutaneous melanoma. Front Oncol. (2022) 12:975255. doi: 10.3389/fonc.2022.975255 36059641 PMC9434152

[74] Christodoulou MI Zaravinos A. Single-cell analysis in immuno-oncology. Int J Mol Sci. (2023) 24:8422. doi: 10.3390/ijms24098422 37176128 PMC10178969

[75] Reza KK Dey S Wuethrich A Jing W Behren A Antaw F. *In situ* single cell proteomics reveals circulating tumor cell heterogeneity during treatment. ACS Nano. (2021) 15:11231–43. doi: 10.1021/acsnano.0c10008 34225455

[76] Ding L Sun L Bu MT Zhang Y Scott LN Prins RM. Antigen presentation by clonally diverse CXCR5+ B cells to CD4 and CD8 T cells is associated with durable response to immune checkpoint inhibitors. Front Immunol. (2023) 14:1176994. doi: 10.3389/fimmu.2023.1176994 37435085 PMC10330698

